# Approximating Ground States by Neural Network Quantum States

**DOI:** 10.3390/e21010082

**Published:** 2019-01-17

**Authors:** Ying Yang, Chengyang Zhang, Huaixin Cao

**Affiliations:** 1 School of Mathematics and Information Science, Shaanxi Normal University, Xi’an 710119, China; 2 School of Mathematics and Information Technology, Yuncheng University, Yuncheng 044000, China

**Keywords:** approximation, ground state, neural network quantum state

## Abstract

Motivated by the Carleo’s work (Science, 2017, 355: 602), we focus on finding the neural network quantum statesapproximation of the unknown ground state of a given Hamiltonian *H* in terms of the best relative error and explore the influences of sum, tensor product, local unitary of Hamiltonians on the best relative error. Besides, we illustrate our method with some examples.

## 1. Introduction

The quantum many-body problem is a general name for a vast category of physical problems pertaining to the properties of microscopic systems made of a large number of interacting particles. In such a quantum system, the repeated interactions between particles create quantum correlations [1,2,3], quantum entanglement [4,5,6], Bell nonlocality [7,8,9], Einstein-Poldolsky-Rosen (EPR) steering [10,11,12]. As a consequence, the wave function of the system is a complicated object holding a large amount of information, which usually makes exact or analytical calculations impractical or even impossible. Thus, many-body theoretical physics most often relies on a set of approximations specific to the problem at hand, and ranks among the most computationally intensive fields of science. Gordon [13] indicated that if one knows an accurate energy for the ground state (say from experiment), then one can construct a sequence of upper and lower bounds to the overlap between the approximate function and the true (but unknown) ground-state wave function. Wang [14] constructed a set of intermediate resolvents from the intermediate Hamiltonians introduced by Weinstein. From these intermediate resolvents they obtained a new formula for the lower bound of the overlap between the approximate and exact wave functions of a quantum-mechanical system. Merkel [15] proposed a method and tested it for approximating the integrals over $H^{2}$ and $H^{3}$ required by Weinhold’s technique as products of integrals involving only *H*. Cioslowski [16] constructed a connected-moments expansion for the overlap between the approximate and the exact (but unknown) wave function of the ground state. Hornik [17] shown that LRV’s algorithm, with some relatively trivial modifications, can estimate the overlap of an approximate with the exact wave function. Marmorino [18] derived two methods from the *t* expansion of Horn and Weinstein to bound from above the magnitude of the overlap of an approximate wavefunction with the ground state. Nomura [19] developed a machine learning method to construct accurate ground-state wave functions of strongly interacting and entangled quantum spin as well as fermionic models on lattices.

Artificial neural networks are important tools in machine learning due to their efficient approximation ability [20,21,22,23,24]. Especially, Roux [25] proved that restricted Boltzmann machines are universal approximators of discrete distributions.

Applying neural networks in solving the quantum many-body problem, Carleo and Troyer in [26] demonstrated the remarkable power of a reinforcement learning approach in calculating the ground state or simulating the unitary time evolution of complex quantum systems with strong interactions. Their idea consists in using neural networks as variational wave functions to approximate ground states of many-body quantum systems. In this direction, the networks are trained or optimized by the standard variational Monte Carlo method while a few different neural-network architectures were tested [26,27,28,29], and the most promising results so far have been achieved with Boltzmann machines [29]. In particular, state-of-the-art numerical results have been obtained on popular models with restricted Boltzmann machines (RBM), and recent effort has demonstrated the power of deep Boltzmann machines to represent ground states of many-body Hamiltonians with polynomial-size gap and quantum states generated by any polynomial size quantum circuits [30,31]. Deng et al. [32] show that the RBM can be used to describe topological states and constructed exact representations for symmetry-protected topological states and intrinsic topologically ordered states. Glasser et al. [33] show that there are strong connections between neural network quantum states in the form of RBM and some classes of tensor-network states in arbitrary dimensions and obtain that neural network quantum states and their string-bond-state extension can describe a lattice fractional quantum Hall state exactly. Gardas, Rams and Dziarmaga [34] show that the approach of Carleo and Troyer [26] can be realized experimentally using quantum annealers and they conducted experimental simulations of many-body quantum systems using a hybrid classical-quantum algorithm. Cai and Liu in [35] demonstrated the expressibility of artificial neural networks in quantum many-body physics by showing that a feed-forward neural network with a small number of hidden layers can be trained to approximate with high precision the ground states of some notable quantum many-body systems. In [36], Saito and Kato developed a variational method to obtain many-body ground states of the Bose-Hubbard model using feed forward artificial neural networks and proved that many-body ground states with different numbers of atoms can be generated by a single network. By employing the formalism of tensor networks, Clark [37] show that neural network quantum states given in [26] are a special form of correlator product states.

Despite such exciting developments, it is unknown whether a general state can be expressed by neural networks efficiently. Recently, by generalizing the idea of [26], we introduced in [38] neural networks quantum states (NNQSs) based on general input observables and explored some related properties about NNQSs. Secondly, we established some necessary and sufficient conditions for a general graph state to be represented by an NNQS.

In this paper, based on the NNQSs introduced in [38], we focus on finding the NNQS approximation of the unknown ground state of a given Hamiltonian *H*. The remaining part of this paper is organized as follows. In Section 2, we recall the concept and the related properties of NNQSs introduced in [38]. In Section 3, we explore the NNQS approximation of the unknown ground state of a given Hamiltonian *H* in terms of the best relative error and consider the influence of sum, tensor product, local unitary of Hamiltonian on the best relative error. Besides, we illustrate our method with some examples.

## 2. Neural Network Quantum States

To start with, let us recall the concept and the related properties of NNQSs introduced in [38]. Let $Q_{1},Q_{2},\ldots ,Q_{N}$ be *N* quantum systems with state spaces $H_{1},H_{2},\ldots ,H_{N}$ of dimensions $d_{1},d_{2},\ldots ,d_{N}$, respectively. We consider the composite system *Q* of $Q_{1},Q_{2},\ldots ,Q_{N}$ with state space $H:=H_{1}⊗H_{2}⊗\ldots ⊗H_{N}$.

Let $S_{1},S_{2},\ldots ,S_{N}$ be non-degenerate observables of systems $Q_{1},Q_{2},\ldots ,Q_{N}$, respectively. Then $S=S_{1}⊗S_{2}⊗\ldots ⊗S_{N}$ is an observable of the composite system *Q*. Use $\{|\psi_{k_{j}}\rangle {\}}_{k_{j}=0}^{d_{j}-1}$ to denote the eigenbasis of $S_{j}$ corresponding to eigenvalues ${\left\{\lambda_{k_{j}}\right\}}_{k_{j}=0}^{d_{j}-1}$. Thus,

(1) $$\begin{array}{c} S_{j}|\psi_{k_{j}}\rangle =\lambda_{k_{j}}|\psi_{k_{j}}\rangle \left(k_{j}=0,1,\ldots ,d_{j}-1\right). \end{array}$$

It is easy to check that the eigenvalues and corresponding eigenbasis of $S=S_{1}⊗S_{2}⊗\ldots ⊗S_{N}$ are

(2) $$\begin{array}{c} \lambda_{k_{1}}\lambda_{k_{2}}\ldots \lambda_{k_{N}}\;\mathrm{and}|\psi_{k_{1}}\rangle ⊗|\psi_{k_{2}}\rangle ⊗\ldots ⊗|\psi_{k_{N}}\rangle \left(k_{j}=0,1,\ldots ,d_{j}-1\right), \end{array}$$

respectively. Put

$$V\left(S\right)=\left\{\Lambda_{k_{1}k_{2}\ldots k_{N}}\equiv {\left(\lambda_{k_{1}},\lambda_{k_{2}},\ldots ,\lambda_{k_{N}}\right)}^{T}:k_{j}=0,1,\ldots ,d_{j}-1\right\},$$

called an input space. For parameters

$$a={\left(a_{1},a_{2},\ldots ,a_{N}\right)}^{T}\in C^{N},b={\left(b_{1},b_{2},\ldots ,b_{M}\right)}^{T}\in C^{M},W=\left[W_{ij}\right]\in C^{M\times N},$$

write Ω=(a,b,W) and put

(3) $$\begin{array}{c} \Psi_{S,\Omega }\left(\lambda_{k_{1}},\lambda_{k_{2}},\ldots ,\lambda_{k_{N}}\right)=\sum_{h_{i}=\pm 1}\exp\left(\sum_{j=1}^{N}a_{j}\lambda_{k_{j}}+\sum_{i=1}^{M}b_{i}h_{i}+\sum_{i=1}^{M}\sum_{j=1}^{N}W_{ij}h_{i}\lambda_{k_{j}}\right). \end{array}$$

**Figure 1:**
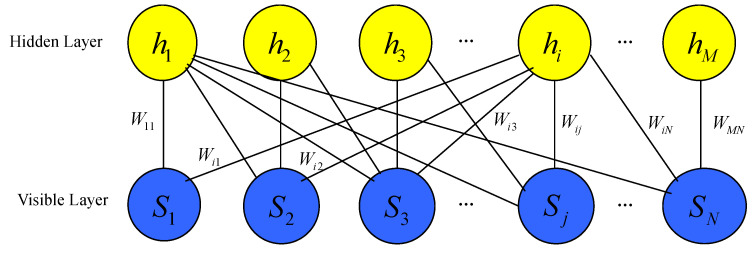
Artificial neural network encoding an NNQS. It is a restricted Boltzmann machine architecture that features a set of *N* visible artificial neurons (blue disks) and a set of *M* hidden neurons (yellow disks). For each value $\Lambda_{k_{1}k_{2}\ldots k_{N}}$ of the input observable *S*, the neural network computes the value of the $\Psi_{S,\Omega }\left(\lambda_{k_{1}},\lambda_{k_{2}},\ldots ,\lambda_{k_{N}}\right)$.

Then we obtain a complex-valued function $\Psi_{S,\Omega }\left(\lambda_{k_{1}},\lambda_{k_{2}},\ldots ,\lambda_{k_{N}}\right)$ of the input variable $\Lambda_{k_{1}k_{2}\ldots k_{N}}$. We call it a *neural network quantum wave function* (NNQWF). Then we define

(4) $$\begin{array}{c} |\Psi_{S,\Omega }\rangle =\sum_{\Lambda_{k_{1}k_{2}\ldots k_{N}}\in V\left(S\right)}\Psi_{S,\Omega }\left(\lambda_{k_{1}},\lambda_{k_{2}},\ldots ,\lambda_{k_{N}}\right)|\psi_{k_{1}}\rangle ⊗|\psi_{k_{2}}\rangle ⊗\ldots ⊗|\psi_{k_{N}}\rangle , \end{array}$$

which is a nonzero vector (not necessarily normalized) of the Hilbert space H. We call it a *neural network quantum state* (NNQS) induced by the parameter Ω=(a,b,W) and the input observable $S=S_{1}⊗S_{2}⊗\ldots ⊗S_{N}$ (Figure 1).

Note that we do not assume that an NNQWF satisfies the normalization condition:

$$\sum_{\Lambda_{k_{1}k_{2}\ldots k_{N}}}{\left|\Psi_{S,\Omega }\left(\lambda_{k_{1}},\lambda_{k_{2}},\ldots ,\lambda_{k_{N}}\right)\right|}^{2}=1.$$

Indeed, when a state can be written as NNQS, this normalization condition is automatically satisfied.

By definition, an NNQWF can be reduced to

(5) $$\begin{array}{c} \Psi_{S,\Omega }\left(\lambda_{k_{1}},\lambda_{k_{2}},\ldots ,\lambda_{k_{N}}\right)=\prod_{j=1}^{N}e^{a_{j}\lambda_{k_{j}}}\cdot \prod_{i=1}^{M}2\cosh\left(b_{i}+\sum_{j=1}^{N}W_{ij}\lambda_{k_{j}}\right). \end{array}$$

**Figure 2:**
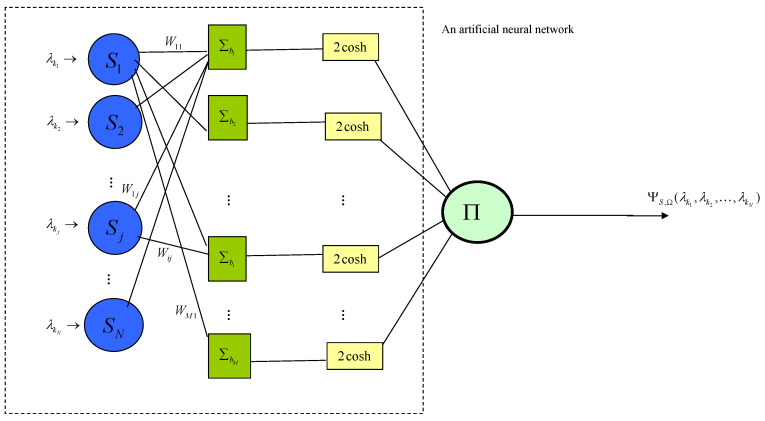
Quantum artificial neural network with parameter Ω=(0,b,W).

It is can be described by the following “quantum artificial neural network” (Figure 2) where $a=0,\;2\cosh\left(z\right)=e^{z}+e^{-z}$, $\sum_{b_{i}}$ and Π are functions such that

$$\sum_{b_{i}}\left(x_{1},x_{2},\ldots ,x_{N}\right)=b_{i}+\sum_{j=1}^{N}x_{j},\;\Pi \left(y_{1},y_{2},\ldots ,y_{M}\right)=\Pi_{i=1}^{M}y_{i}.$$

We call this network a quantum artificial neural network because its input eigenvalues of quantum observables and the outcomes are values of an NNQWF, while it has a network structure similar to a usual artificial neural network.

Next, let us consider the tensor product of the two NNQSs. We have proved the following.

**Proposition** **1.** *[38] Suppose that $|\Psi_{S^{\prime },\Omega^{\prime }}^{\prime }\rangle$ and $|\Psi_{S^{\prime \prime },\Omega^{\prime \prime }}^{\prime \prime }\rangle$ are two NNQSs with parameters*

$$S^{\prime }=S_{1}^{\prime }⊗\ldots ⊗S_{N^{\prime }}^{\prime },S^{\prime \prime }=S_{1}^{\prime \prime }⊗\ldots ⊗S_{N^{\prime \prime }}^{\prime \prime },\Omega^{\prime }=\left(a^{\prime },b^{\prime },W^{\prime }\right),\Omega^{\prime \prime }=\left(a^{\prime \prime },b^{\prime \prime },W^{\prime \prime }\right),$$

*respectively. Then $|\Psi_{S^{\prime },\Omega^{\prime }}^{\prime }\rangle ⊗|\Psi_{S^{\prime \prime },\Omega^{\prime \prime }}^{\prime \prime }\rangle$ is also an NNQS $|\Phi_{S,\Omega }\rangle$ with parameters*

$$S=S^{\prime }⊗S^{\prime \prime },\Omega =\left(a,b,W\right),N=N^{\prime }+N^{\prime \prime },M=M^{\prime }+M^{\prime \prime },$$

$$a=\left(\begin{array}{c} a^{\prime }\\ a^{\prime \prime } \end{array}\right),b=\left(\begin{array}{c} b^{\prime }\\ b^{\prime \prime } \end{array}\right),W=\left[W_{ij}\right]=\left(\begin{array}{cc} W_{M^{\prime }\times N^{\prime }}^{\prime } & 0\\ 0 & W_{M^{\prime \prime }\times N^{\prime \prime }}^{\prime \prime } \end{array}\right).$$

Now, we discuss the influence of local unitary operation (LUO) on an NNQS. We conclude this as follows.

**Proposition** **2.** *[38] Suppose that $|\Psi_{S,\Omega }\rangle$ is an NNQS and $U=U_{1}⊗U_{2}⊗\ldots ⊗U_{N}$ is a local unitary operator on H. Then $U|\Psi_{S,\Omega }\rangle =|\Psi_{USU^{†},\Omega }\rangle$, which is also an NNQS with the input observable $USU^{†}$ and the parameter *Ω*, and has the same NNQWF as $|\Psi_{S,\Omega }\rangle$.*

**Remark** **1.** *It can be seen from Proposition 2 that if two pure states are LU-equivalent and an NNQS representation of one of the two states is easily given, then that of another state can be obtained from that of the former.*

To conclude this section, we discuss a special class of NNQSs.

When $S=\sigma_{1}^{z}⊗\sigma_{2}^{z}⊗\ldots ⊗\sigma_{N}^{z}$, we have

$$\lambda_{k_{j}}=\left\{\begin{array}{cc} 1, & k_{j}=0\\ -1, & k_{j}=1 \end{array}\right.,|\psi_{k_{j}}\rangle =\left\{\begin{array}{cc} |0\rangle , & k_{j}=0\\ |1\rangle , & k_{j}=1 \end{array}\right.\left(1\leq j\leq N\right),$$

and $V\left(S\right)={\left\{1,-1\right\}}^{N}$.

In this case, the NNQS (4) becomes

(6) $$\begin{array}{c} |\Psi_{S,\Omega }\rangle =\sum_{\Lambda_{k_{1}k_{2}\ldots k_{N}}\in {\left\{1,-1\right\}}^{N}}\Psi_{S,\Omega }\left(\lambda_{k_{1}},\lambda_{k_{2}},\ldots ,\lambda_{k_{N}}\right)|\psi_{k_{1}}\rangle ⊗|\psi_{k_{2}}\rangle ⊗\ldots ⊗|\psi_{k_{N}}\rangle . \end{array}$$

This leads to the NNQS induced in [26] and discussed in [32]. We call such an NNQS a *spin-z NNQS*.

## 3. Approximating Ground States by Neural Network Quantum States

In this section, we try to find approximate solution to the static Schrödinger equation H|ψ〉=E|ψ〉 for a given Hamiltonian *H*. For example, to find approximation of ground states by neural network quantum states.

Let $|\Psi_{S,\Omega }\rangle$ be an NNQS given by Equation (4) and let *H* be a Hamiltonian whose smallest eigenvalue $E_{exact}$ is not zero. Put

$$E_{H}\left(S,\Omega \right)=\frac{\langle \Psi_{S,\Omega }|H|\Psi_{S,\Omega }\rangle }{\langle \Psi_{S,\Omega }|\Psi_{S,\Omega }\rangle }.$$

We seek the minimum relative error between $E_{H}\left(S,\Omega \right)$ and $E_{exact}$ over Ω,

(7) $$\begin{array}{c} \epsilon =\min_{\Omega }\frac{|E_{H}\left(S,\Omega \right)-E_{exact}|}{|E_{exact}|}. \end{array}$$

We call ϵ the *best relative error* between $E_{H}\left(S,\Omega \right)$ and $E_{exact}$.

Obviously, when the minimum in Equation (7) is attained at the parameter Ω, we can use the normalized NNQS ${\langle \Psi_{S,\Omega }|\Psi_{S,\Omega }\rangle }^{-\frac{1}{2}}|\Psi_{S,\Omega }\rangle$ as an approximation of the ground state of *H* with the best relative error ϵ.

Generally, $E_{H}\left(S,\Omega \right)\geq E_{exact}$. Hence, ϵ can also be expressed as

$$\epsilon =\min_{\Omega }\frac{E_{H}\left(S,\Omega \right)-E_{exact}}{|E_{exact}|}.$$

Next, we discuss the influence of the sum of Hamiltonians on the best relative error. We obtain the following conclusion.

**Proposition** **3.** *Suppose that $H_{1}$ and $H_{2}$ are two Hamiltonians, $E_{exact}^{\prime }$, $E_{exact}^{\prime \prime }$ and $E_{exact}$ are the smallest eigenvalue of $H_{1}$, $H_{2}$ and $H_{1}+H_{2}$, respectively, $|\Psi_{S,\Omega }\rangle$ is an NNQS. Then*

$$E_{H_{1}+H_{2}}\left(S,\Omega \right)=E_{H_{1}}\left(S,\Omega \right)+E_{H_{2}}\left(S,\Omega \right).$$

*Furthermore, if $\min_{\Omega }\left(E_{H_{1}}\left(S,\Omega \right)+E_{H_{2}}\left(S,\Omega \right)\right)=\min_{\Omega }E_{H_{1}}\left(S,\Omega \right)+\min_{\Omega }E_{H_{2}}\left(S,\Omega \right)$, then*

$$0\leq \epsilon \leq \epsilon_{1}+\epsilon_{2},$$

*where*

$$\epsilon_{1}=\min_{\Omega }\frac{|E_{H_{1}}\left(S,\Omega \right)-E_{exact}^{\prime }|}{|E_{exact}^{\prime }|},\;\epsilon_{2}=\min_{\Omega }\frac{|E_{H_{2}}\left(S,\Omega \right)-E_{exact}^{\prime \prime }|}{|E_{exact}^{\prime \prime }|},\;\epsilon =\min_{\Omega }\frac{|E_{H_{1}+H_{2}}\left(S,\Omega \right)-E_{exact}|}{|E_{exact}|}.$$

**Proof.**  We can easily compute that

$$\begin{array}{ccc} E_{H_{1}+H_{2}}\left(S,\Omega \right) & = & \frac{\langle \Psi_{S,\Omega }|H_{1}+H_{2}|\Psi_{S,\Omega }\rangle }{\langle \Psi_{S,\Omega }|\Psi_{S,\Omega }\rangle }\\ & = & \frac{\langle \Psi_{S,\Omega }|H_{1}|\Psi_{S,\Omega }\rangle }{\langle \Psi_{S,\Omega }|\Psi_{S,\Omega }\rangle }+\frac{\langle \Psi_{S,\Omega }|H_{2}|\Psi_{S,\Omega }\rangle }{\langle \Psi_{S,\Omega }|\Psi_{S,\Omega }\rangle }\\ & = & E_{H_{1}}\left(S,\Omega \right)+E_{H_{2}}\left(S,\Omega \right). \end{array}$$

It is easily see that ϵ≥0. Generally,

$$\min_{\Omega }E_{H_{1}}\left(S,\Omega \right)\geq E_{exact}^{\prime },\;\min_{\Omega }E_{H_{2}}\left(S,\Omega \right)\geq E_{exact}^{\prime \prime },\;\min_{\Omega }E_{H_{1}+H_{2}}\left(S,\Omega \right)\geq E_{exact}.$$

Besides, when $\min_{\Omega }\left(E_{H_{1}}\left(S,\Omega \right)+E_{H_{2}}\left(S,\Omega \right)\right)=\min_{\Omega }E_{H_{1}}\left(S,\Omega \right)+\min_{\Omega }E_{H_{2}}\left(S,\Omega \right)$, we see from $E_{exact}\geq E_{exact}^{\prime }+E_{exact}^{\prime \prime }$ that

$$\begin{array}{ccc} \epsilon & = & \frac{|\min_{\Omega }E_{H_{1}+H_{2}}\left(S,\Omega \right)-E_{exact}|}{|E_{exact}|}\\ & = & \frac{|\min_{\Omega }\left(E_{H_{1}}\left(S,\Omega \right)+E_{H_{2}}\left(S,\Omega \right)\right)-E_{exact}|}{|E_{exact}|}\\ & \leq & \frac{|\min_{\Omega }E_{H_{1}}\left(S,\Omega \right)+\min_{\Omega }E_{H_{2}}\left(S,\Omega \right)-E_{exact}^{\prime }-E_{exact}^{\prime \prime }|}{|E_{exact}^{\prime }+E_{exact}^{\prime \prime }|}\\ & \leq & \frac{|\min_{\Omega }E_{H_{1}}\left(S,\Omega \right)-E_{exact}^{\prime }|}{|E_{exact}^{\prime }|}+\frac{|\min_{\Omega }E_{H_{2}}\left(S,\Omega \right)-E_{exact}^{\prime \prime }|}{|E_{exact}^{\prime \prime }|}\\ & = & \epsilon_{1}+\epsilon_{2}. \end{array}$$

 □

Now, we discuss the influence of tensor product of Hamiltonians on the best relative error. We get the following conclusion.

**Proposition** **4.** *Suppose that $H_{1}$ and $H_{2}$ are two Hamiltonians, $E_{exact}^{\prime }$, $E_{exact}^{\prime \prime }$ and $E_{exact}$ are the smallest eigenvalue of $H_{1}$, $H_{2}$ and $H_{1}⊗H_{2}$, respectively. $|\Psi_{S^{\prime },\Omega^{\prime }}^{\prime }\rangle$ and $|\Psi_{S^{\prime \prime },\Omega^{\prime \prime }}^{\prime \prime }\rangle$ are two NNQSs with parameters*

$$S^{\prime }=S_{1}^{\prime }⊗\ldots ⊗S_{N^{\prime }}^{\prime },S^{\prime \prime }=S_{1}^{\prime \prime }⊗\ldots ⊗S_{N^{\prime \prime }}^{\prime \prime },\Omega^{\prime }=\left(a^{\prime },b^{\prime },W^{\prime }\right),\Omega^{\prime \prime }=\left(a^{\prime \prime },b^{\prime \prime },W^{\prime \prime }\right),$$

*respectively. Let*

$$S_{0}=S^{\prime }⊗S^{\prime \prime },\Omega_{0}=\left(a_{0},b_{0},W_{0}\right),N=N^{\prime }+N^{\prime \prime },M_{0}=M^{\prime }+M^{\prime \prime },$$

$$a_{0}=\left(\begin{array}{c} a^{\prime }\\ a^{\prime \prime } \end{array}\right),b_{0}=\left(\begin{array}{c} b^{\prime }\\ b^{\prime \prime } \end{array}\right),W_{0}=\left[W_{ij}\right]=\left(\begin{array}{cc} W_{M^{\prime }\times N^{\prime }}^{\prime } & 0\\ 0 & W_{M^{\prime \prime }\times N^{\prime \prime }}^{\prime \prime } \end{array}\right).$$

*Then*

$$E_{H_{1}⊗H_{2}}\left(S_{0},\Omega_{0}\right)=E_{H_{1}}\left(S^{\prime },\Omega^{\prime }\right)\cdot E_{H_{2}}\left(S^{\prime \prime },\Omega^{\prime \prime }\right).$$

*Furthermore, if $H_{1}$ and $H_{2}$ are positive definite, then $\epsilon^{\prime }\epsilon^{\prime \prime }\leq \epsilon^{0}$ where*

$$\epsilon^{\prime }=\min_{\Omega^{\prime }}\frac{|E_{H_{1}}\left(S^{\prime },\Omega^{\prime }\right)-E_{exact}^{\prime }|}{|E_{exact}^{\prime }|},\;\epsilon^{\prime \prime }=\min_{\Omega^{\prime \prime }}\frac{|E_{H_{2}}\left(S^{\prime \prime },\Omega^{\prime \prime }\right)-E_{exact}^{\prime \prime }|}{|E_{exact}^{\prime \prime }|},$$

$$\epsilon^{0}=\min_{\Omega_{0}}\frac{|E_{H_{1}⊗H_{2}}\left(S_{0},\Omega_{0}\right)-E_{exact}|}{|E_{exact}|}.$$

**Proof.**  Since $|\Psi_{S^{\prime },\Omega^{\prime }}^{\prime }\rangle$ and $|\Psi_{S^{\prime \prime },\Omega^{\prime \prime }}^{\prime \prime }\rangle$ are two NNQSs, we know from Proposition 1 that $|\Psi_{S^{\prime },\Omega^{\prime }}^{\prime }\rangle ⊗|\Psi_{S^{\prime \prime },\Omega^{\prime \prime }}^{\prime \prime }\rangle =|\Phi_{S_{0},\Omega_{0}}\rangle$ is also an NNQS. Furthermore, we can compute

$$\begin{array}{ccc} E_{H_{1}⊗H_{2}}\left(S_{0},\Omega_{0}\right) & = & \frac{\langle \Psi_{S_{0},\Omega_{0}}|H_{1}⊗H_{2}|\Psi_{S_{0},\Omega_{0}}\rangle }{\langle \Psi_{S_{0},\Omega_{0}}|\Psi_{S_{0},\Omega_{0}}\rangle }\\ & = & \frac{\langle \Psi_{S^{\prime },\Omega^{\prime }}|H_{1}|\Psi_{S^{\prime },\Omega^{\prime }}\rangle }{\langle \Psi_{S^{\prime },\Omega^{\prime }}|\Psi_{S^{\prime },\Omega^{\prime }}\rangle }\cdot \frac{\langle \Psi_{S^{\prime \prime },\Omega^{\prime \prime }}|H_{2}|\Psi_{S^{\prime \prime },\Omega^{\prime \prime }}\rangle }{\langle \Psi_{S^{\prime \prime },\Omega^{\prime \prime }}|\Psi_{S^{\prime \prime },\Omega^{\prime \prime }}\rangle }\\ & = & E_{H_{1}}\left(S^{\prime },\Omega^{\prime }\right)\cdot E_{H_{2}}\left(S^{\prime \prime },\Omega^{\prime \prime }\right). \end{array}$$

Since $H_{1}$ and $H_{2}$ are positive, $E_{exact}=E_{exact}^{\prime }E_{exact}^{\prime \prime }$. Observe that

$$\min_{\Omega^{\prime }}E_{H_{1}}\left(S^{\prime },\Omega^{\prime }\right)\geq E_{exact}^{\prime }>0,\;\min_{\Omega^{\prime \prime }}E_{H_{2}}\left(S^{\prime \prime },\Omega^{\prime \prime }\right)\geq E_{exact}^{\prime \prime }>0,\;\min_{\Omega_{0}}E_{H_{1}⊗H_{2}}\left(S_{0},\Omega_{0}\right)\geq E_{exact}>0.$$

Thus, we have

$$\begin{array}{ccc} \epsilon^{0} & = & \frac{|\min_{\Omega_{0}}E_{H_{1}⊗H_{2}}\left(S_{0},\Omega_{0}\right)-E_{exact}|}{|E_{exact}|}\\ & = & \frac{|\min_{\Omega^{\prime }}E_{H_{1}}\left(S^{\prime },\Omega^{\prime }\right)\cdot \min_{\Omega^{\prime \prime }}E_{H_{2}}\left(S^{\prime \prime },\Omega^{\prime \prime }\right)-E_{exact}^{\prime }E_{exact}^{\prime \prime }|}{|E_{exact}^{\prime }\left|\cdot \right|E_{exact}^{\prime \prime }|}\\ & \geq & \frac{|\min_{\Omega^{\prime }}E_{H_{1}}\left(S^{\prime },\Omega^{\prime }\right)-E_{exact}^{\prime }|}{|E_{exact}^{\prime }|}\cdot \frac{|\min_{\Omega^{\prime \prime }}E_{H_{2}}\left(S^{\prime \prime },\Omega^{\prime \prime }\right)-E_{exact}^{\prime \prime }|}{|E_{exact}^{\prime \prime }|}\\ & = & \epsilon^{\prime }\epsilon^{\prime \prime }. \end{array}$$

 □

Now, we discuss the influence of local unitary operation on the best relative error. We conclude this conclusion as follows.

**Proposition** **5.** *Suppose that H is a Hamiltonian, $|\Psi_{S,\Omega }\rangle$ is an NNQS and $U=U_{1}⊗U_{2}⊗\ldots ⊗U_{N}$ is a local unitary operator on H. $E_{exact},E_{exact}^{\prime }$ are the smallest eigenvalue of H and $UHU^{†}$, respectively. Then*

$$E_{UHU^{†}}\left(S,\Omega \right)=E_{H}\left(U^{†}SU,\Omega \right),$$

*and $\epsilon =\epsilon^{\prime }$ where*

$$\epsilon =\min_{\Omega }\frac{|E_{H}\left(U^{†}SU,\Omega \right)-E_{exact}|}{|E_{exact}|},\;\epsilon^{\prime }=\min_{\Omega }\frac{|E_{UHU^{†}}\left(S,\Omega \right)-E_{exact}^{\prime }|}{|E_{exact}^{\prime }|}.$$

**Proof.**  We can obtain from Proposition 2 that $U^{†}|\Psi_{S,\Omega }\rangle =|\Psi_{U^{†}SU,\Omega }\rangle$, which is also an NNQS. Therefore

$$\begin{array}{ccc} E_{UHU^{†}}\left(S,\Omega \right) & = & \frac{\langle \Psi_{S,\Omega }|UHU^{†}|\Psi_{S,\Omega }\rangle }{\langle \Psi_{S,\Omega }|\Psi_{S,\Omega }\rangle }\\ & = & \frac{\langle \Psi_{U^{†}SU,\Omega }|H|\Psi_{U^{†}SU,\Omega }\rangle }{\langle \Psi_{U^{†}SU,\Omega }|\Psi_{U^{†}SU,\Omega }\rangle }\\ & = & E_{H}\left(U^{†}SU,\Omega \right). \end{array}$$

Since *U* is a local unitary operator, $E_{exact}=E_{exact}^{\prime }$. We can easily obtain that $\epsilon =\epsilon^{\prime }$. □

Lastly, we give two examples in order to illustrate our method.

**Example** **1.** *Suppose that H=|00〉〈00|+2|01〉〈01|+3|10〉〈10|+4|11〉〈11|. Then H can be represented under the basis {|00〉,|01〉,|10〉,|11〉} by H=diag(1,2,3,4). It is easy to see that the minimum eigenvalue of H is *1*, the ground state is |00〉.*

*Next we use spin-z NNQSs*

$$\begin{array}{c} |\Psi_{S,\Omega }\rangle =\sum_{\Lambda_{k_{1}k_{2}}\in {\left\{1,-1\right\}}^{2}}\Psi_{S,\Omega }\left(\lambda_{k_{1}},\lambda_{k_{2}}\right)|\psi_{k_{1}}\rangle ⊗|\psi_{k_{2}}\rangle . \end{array}$$

*to approximate the ground state |00〉 of H, where*

$$\begin{array}{c} \Psi_{S,\Omega }\left(\lambda_{k_{1}},\lambda_{k_{2}}\right)=\prod_{j=1}^{2}e^{a_{j}\lambda_{k_{j}}}\cdot \prod_{i=1}^{M}2\cosh\left(b_{i}+\sum_{j=1}^{2}W_{ij}\lambda_{k_{j}}\right). \end{array}$$

*When N=M=2, we have*

$$\begin{array}{ccc} |\Psi_{S,\Omega }\rangle & = & 4e^{a_{1}}e^{a_{2}}\cosh\left(b_{1}+W_{11}+W_{12}\right)\cosh\left(b_{2}+W_{21}+W_{22}\right)|00\rangle \\ & + & 4e^{a_{1}}e^{-a_{2}}\cosh\left(b_{1}+W_{11}-W_{12}\right)\cosh\left(b_{2}+W_{21}-W_{22}\right)|01\rangle \\ & + & 4e^{-a_{1}}e^{a_{2}}\cosh\left(b_{1}-W_{11}+W_{12}\right)\cosh\left(b_{2}-W_{21}+W_{22}\right)|10\rangle \\ & + & 4e^{-a_{1}}e^{-a_{2}}\cosh\left(b_{1}-W_{11}-W_{12}\right)\cosh\left(b_{2}-W_{21}-W_{22}\right)|11\rangle . \end{array}$$

*We can easily calculate that*

$$\begin{array}{ccc} E_{H}\left(S,\Omega \right) & = & \left({\left|e^{a_{1}}e^{a_{2}}\cosh\left(b_{1}+W_{11}+W_{12}\right)\cosh\left(b_{2}+W_{21}+W_{22}\right)\right|}^{2}\right.\\ & + & \left.2{\left|e^{a_{1}}e^{-a_{2}}\cosh\left(b_{1}+W_{11}-W_{12}\right)\cosh\left(b_{2}+W_{21}-W_{22}\right)\right|}^{2}\right.\\ & + & \left.3{\left|e^{-a_{1}}e^{a_{2}}\cosh\left(b_{1}-W_{11}+W_{12}\right)\cosh\left(b_{2}-W_{21}+W_{22}\right)\right|}^{2}\right.\\ & + & \left.4{\left|e^{-a_{1}}e^{-a_{2}}\cosh\left(b_{1}-W_{11}-W_{12}\right)\cosh\left(b_{2}-W_{21}-W_{22}\right)\right|}^{2}\right)\\ & & /\left({\left|e^{a_{1}}e^{a_{2}}\cosh\left(b_{1}+W_{11}+W_{12}\right)\cosh\left(b_{2}+W_{21}+W_{22}\right)\right|}^{2}\right.\\ & + & \left.{\left|e^{a_{1}}e^{-a_{2}}\cosh\left(b_{1}+W_{11}-W_{12}\right)\cosh\left(b_{2}+W_{21}-W_{22}\right)\right|}^{2}\right.\\ & + & \left.{\left|e^{-a_{1}}e^{a_{2}}\cosh\left(b_{1}-W_{11}+W_{12}\right)\cosh\left(b_{2}-W_{21}+W_{22}\right)\right|}^{2}\right.\\ & + & \left.{\left|e^{-a_{1}}e^{-a_{2}}\cosh\left(b_{1}-W_{11}-W_{12}\right)\cosh\left(b_{2}-W_{21}-W_{22}\right)\right|}^{2}\right). \end{array}$$

*Next we seek the minimum value of $E_{H}\left(S,\Omega \right)$ over *Ω*. By letting*

$$b_{1}=x_{1},b_{2}=x_{2},W_{11}=x_{3},W_{12}=x_{4},W_{21}=x_{5},W_{22}=x_{6},a_{1}=x_{7},a_{2}=x_{8},$$

*we define a function g by*

$$\begin{array}{cc} & g(x_{1},x_{2},\ldots ,x_{8})\\ = & ({\left|e^{x_{7}+x_{8}}\cdot \cosh\left(x_{1}+x_{3}+x_{4}\right)\cdot \cosh\left(x_{2}+x_{5}+x_{6}\right)\right|}^{2}\\ & +2{\left|e^{x_{7}-x_{8}}\cdot \cosh\left(x_{1}+x_{3}-x_{4}\right)\cdot \cosh\left(x_{2}+x_{5}-x_{6}\right)\right|}^{2}\\ & +3{\left|e^{-x_{7}+x_{8}}\cdot \cosh\left(x_{1}-x_{3}+x_{4}\right)\cdot \cosh\left(x_{2}-x_{5}+x_{6}\right)\right|}^{2}\\ & +4{\left|e^{-x_{7}-x_{8}}\cdot \cosh\left(x_{1}-x_{3}-x_{4}\right)\cdot \cosh\left(x_{2}-x_{5}-x_{6}\right)\right|}^{2})\\ & /({\left|e^{x_{7}+x_{8}}\cdot \cosh\left(x_{1}+x_{3}+x_{4}\right)\cdot \cosh\left(x_{2}+x_{5}+x_{6}\right)\right|}^{2}\\ & +{\left|e^{x_{7}-x_{8}}\cdot \cosh\left(x_{1}+x_{3}-x_{4}\right)\cdot \cosh\left(x_{2}+x_{5}-x_{6}\right)\right|}^{2}\\ & +{\left|e^{-x_{7}+x_{8}}\cdot \cosh\left(x_{1}-x_{3}+x_{4}\right)\cdot \cosh\left(x_{2}-x_{5}+x_{6}\right)\right|}^{2}\\ & +{\left|e^{-x_{7}-x_{8}}\cdot \cosh\left(x_{1}-x_{3}-x_{4}\right)\cdot \cosh\left(x_{2}-x_{5}-x_{6}\right)\right|}^{2}) \end{array}$$

**Figure 3:**
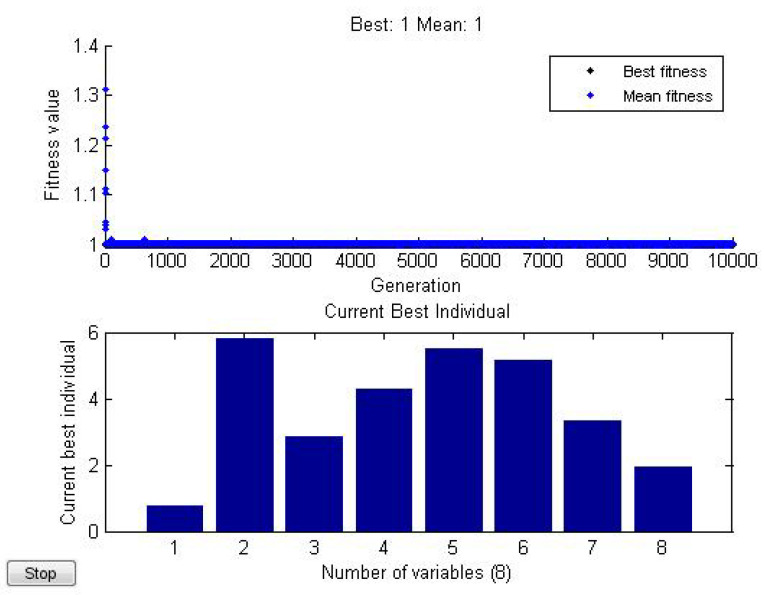
Numerically minimize *g* over $x_{1},x_{2},\ldots ,x_{8}$ by optimization.

*and then numerically minimize g over $x_{1},x_{2},\ldots ,x_{8}$ (see Figure 3).*

*By using Matlab, we find*

$$\begin{array}{c} \min_{x_{i}}g\left(x_{1},x_{2},\ldots ,x_{8}\right)=g\left(0.743,5.788,2.843,4.274,5.501,5.148,3.312,1.916\right)=1. \end{array}$$

*We obtain*

$$\epsilon =\min_{\Omega }\frac{|E_{H}\left(S,\Omega \right)-E_{exact}|}{|E_{exact}|}=0$$

*Meanwhile, the corresponding NNQS is*

$$|\Psi_{S,\Omega }\rangle =6.6458\times 10^{12}|00\rangle +4.6761\times 10^{3}|01\rangle +505.6622|10\rangle +406.2882|11\rangle ,$$

*then the normalized NNQS is*

$$|\Psi_{S,\Omega }^{\prime }\rangle =\frac{|\Psi_{S,\Omega }\rangle }{\sqrt{\langle \Psi_{S,\Omega }|\Psi_{S,\Omega }\rangle }}\approx |00\rangle .$$

*Besides, we can also calculate the distance between the actual ground state |00〉 and the approximate state $|\Psi_{S,\Omega }^{\prime }\rangle$ to be*

$$dist(|00\rangle ,|\Psi_{S,\Omega }^{\prime }\rangle )=∥|00\rangle -|\Psi_{S,\Omega }^{\prime }\rangle ∥\approx 0.$$

**Example** **2.** *Suppose that*

$$H_{N}^{cluster}=-\sum_{i=1}^{N}\sigma_{i-1}^{z}\sigma_{i}^{x}\sigma_{i+1}^{z},$$

*where $\sigma_{0}^{z}=I,\sigma_{N+1}^{z}=I$. It is easy to see that the minimum eigenvalue of $H_{N}^{cluster}$ is −N, the ground state is cluster state $|C_{N}\rangle$. Hence, $E_{exact}=-N$.*

*Next we use spin-z NNQSs*

$$\begin{array}{c} |\Psi_{S,\Omega }\rangle =\sum_{\Lambda_{k_{1}k_{2}\ldots k_{N}}\in {\left\{1,-1\right\}}^{N}}\Psi_{S,\Omega }\left(\lambda_{k_{1}},\lambda_{k_{2}},\ldots ,\lambda_{k_{N}}\right)|\psi_{k_{1}}\rangle ⊗|\psi_{k_{2}}\rangle ⊗\ldots ⊗|\psi_{k_{N}}\rangle , \end{array}$$

*to approximate the ground state $|C_{N}\rangle$ of $H_{N}^{cluster}$, where*

$$\begin{array}{c} \Psi_{S,\Omega }\left(\lambda_{k_{1}},\lambda_{k_{2}},\ldots ,\lambda_{k_{N}}\right)=\prod_{j=1}^{N}e^{a_{j}\lambda_{k_{j}}}\cdot \prod_{i=1}^{M}2\cosh\left(b_{i}+\sum_{j=1}^{N}W_{ij}\lambda_{k_{j}}\right). \end{array}$$

*(i) When N=M=2. By letting*

$$a_{1}=x_{1}+x_{2}i,a_{2}=x_{3}+x_{4}i,b_{1}=x_{5}+x_{6}i,b_{2}=x_{7}+x_{8}i,$$

$$W_{11}=x_{9}+x_{10}i,W_{12}=x_{11}+x_{12}i,W_{21}=x_{13}+x_{14}i,W_{22}=x_{15}+x_{16}i,$$

**Figure 4:**
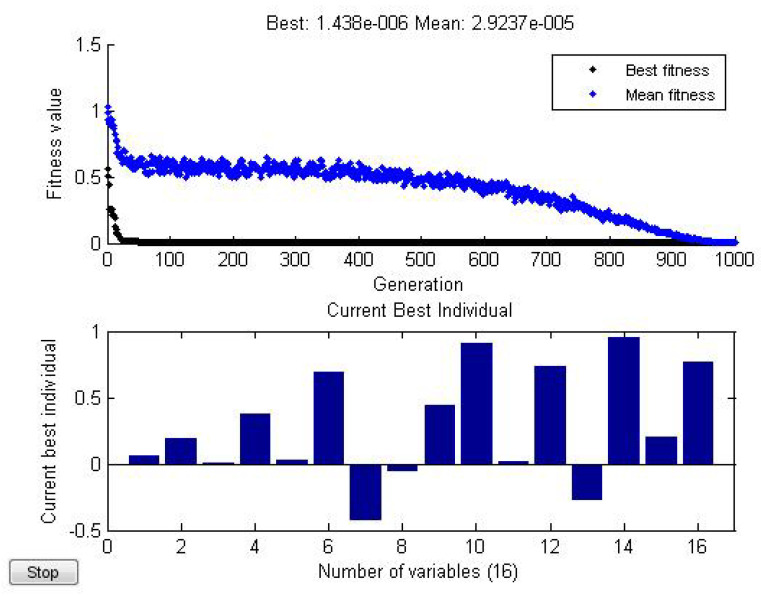
Numerically minimize *ϵ* by optimization.

*using Matlab(see Figure 4), we find*

$$\epsilon =1.438\times 10^{-6},$$

*where*

$$a=\left(\begin{array}{c} 0.065+0.194i\\ 0.008+0.37i \end{array}\right),b=\left(\begin{array}{c} 0.022+0.693i\\ -0.431-0.056i \end{array}\right),W=\left(\begin{array}{cc} 0.437+0.909i & 0.018+0.733i\\ -0.272+0.952i & 0.2+0.771i \end{array}\right).$$

*Meanwhile, the corresponding NNQS is*

$$|\Psi_{S,\Omega }\rangle =\left(3.5877+0.4407i\right)|00\rangle +\left(3.5755+0.5083i\right)|01\rangle +\left(3.5805+0.4372i\right)|10\rangle +\left(-3.5698-0.5169i\right)|11\rangle ,$$

*then normalized NNQS is*

$$\begin{array}{ccc} |\Psi_{S,\Omega }^{\prime }\rangle & = & \frac{|\Psi_{S,\Omega }\rangle }{\sqrt{\langle \Psi_{S,\Omega }|\Psi_{S,\Omega }\rangle }}=\left(0.4969+0.0610i\right)|00\rangle +\left(0.4952+0.0704i\right)|01\rangle \\ & & +(0.4959+0.0606i)|10\rangle +(-0.4944-0.0716i)|11\rangle . \end{array}$$

*Besides, we can also calculate the fidelity between the actual ground state*

$$|C_{2}\rangle =\frac{1}{2}|00\rangle +\frac{1}{2}|01\rangle +\frac{1}{2}|10\rangle -\frac{1}{2}|11\rangle$$

*and the approximate state $|\Psi_{S,\Omega }^{\prime }\rangle$ to be*

$$F(|C_{2}\rangle ,|\Psi_{S,\Omega }^{\prime }\rangle )=|\langle C_{2}|\Psi_{S,\Omega }^{\prime }\rangle |=0.9999\approx 1.$$

*Hence, $|C_{2}\rangle \approx |\Psi_{S,\Omega }^{\prime }\rangle$.*

*In addition, we find that when N=2, ϵ gets smaller and smaller as M changes, see Table 1.*

**Figure 5:**
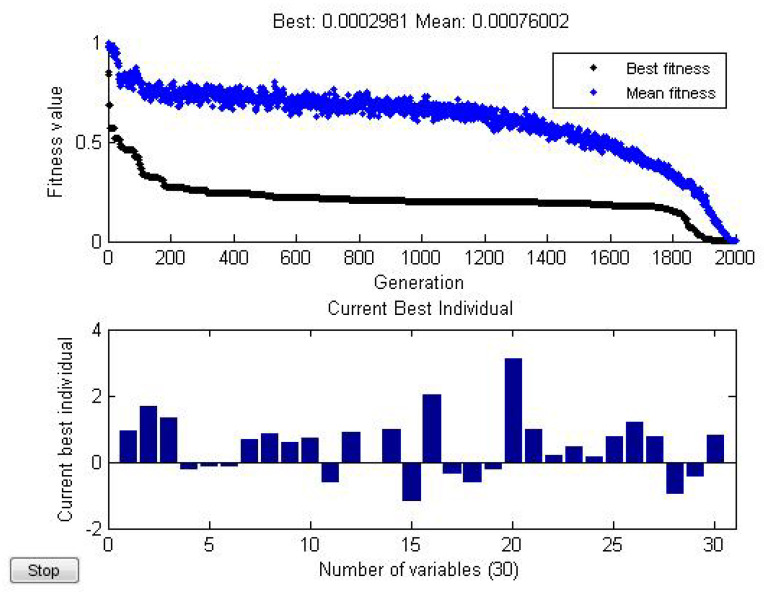
Numerically minimize *ϵ* by optimization.

*(ii) When N=3,M=3. By using Matlab (see Figure 5), we find*

$$\epsilon =2.981\times 10^{-4}.$$

*where*

$$a=\left(\begin{array}{c} 0.956+1.669i\\ 1.309-0.255i\\ -0.148-0.152i \end{array}\right),b=\left(\begin{array}{c} 0.653+0.863i\\ 0.569+0.706i\\ -0.613+0.894i \end{array}\right),$$

$$W=\left(\begin{array}{ccc} -0.066+0.969i & -1.213+2.029i & -0.354-0.647i\\ -0.233+3.12i & 0.986+0.198i & 0.438+0.16i\\ 0.74+1.206i & 0.749-0.985i & -0.445+0.8i \end{array}\right).$$

Meanwhile, the corresponding NNQS is

$$\begin{array}{ccc} |\Psi_{S,\Omega }\rangle & = & (-4.5329-9.8797i)|000\rangle +(-4.4661-9.6734i)|001\rangle +(-4.5709-9.9717i)|010\rangle \\ & & +(4.3557+9.7498i)|011\rangle +(-4.4258-9.8957i)|100\rangle +(-4.4603-9.6152i)|101\rangle \\ & & +(4.6706+9.8781i)|110\rangle +(-4.1489-9.7979i)|111\rangle , \end{array}$$

then normalized NNQS is

$$\begin{array}{ccc} |\Psi_{S,\Omega }^{\prime }\rangle & = & \frac{|\Psi_{S,\Omega }\rangle }{\sqrt{\langle \Psi_{S,\Omega }|\Psi_{S,\Omega }\rangle }}=\left(-0.1488-0.3242i\right)|000\rangle +\left(-0.1466-0.3175i\right)|001\rangle \\ & & +(-0.1500-0.3272i)|010\rangle +(0.1429+0.32i)|011\rangle +(-0.1452-0.3248i)|100\rangle \\ & & +(-0.1464-0.3156i)|101\rangle +(0.1533+0.3242i)|110\rangle +(-0.1362-0.3215i)|111\rangle . \end{array}$$

Besides, we can also calculate the fidelity between the actual ground state

$$|C_{3}\rangle =\frac{1}{2\sqrt{2}}(|000\rangle +|001\rangle +|010\rangle -|011\rangle +|100\rangle +|101\rangle -|110\rangle +\left|111\rangle \right)$$

and the approximate state $|\Psi_{S,\Omega }^{\prime }\rangle$ to be

$$F(|C_{3}\rangle ,|\Psi_{S,\Omega }^{\prime }\rangle )=|\langle C_{3}|\Psi_{S,\Omega }^{\prime }\rangle |=0.9999\approx 1.$$

Hence, $|C_{3}\rangle \approx |\Psi_{S,\Omega }^{\prime }\rangle$.

## 4. Conclusions

In this paper, the question of approximating ground states by neural network quantum states has been discussed in terms of the best relative error (BRE). Some properties of the BREs have been obtained, including the BREs of sums, tensor products, and local unitary transformations of Hamiltonians. Besides, our method has been illustrated with two examples.

## Figures and Tables

**Table 1:** The numerical simulation results of N,M.

*N*	*M*	ϵ
2	2	$1.438\times 10^{-6}$
2	4	$1.0716\times 10^{-6}$
2	6	$6.7887\times 10^{-7}$
2	8	$4.987\times 10^{-7}$
